# The Treatment of Cancer

**Published:** 1904-12-10

**Authors:** 


// The Hospital.

A Journal of

The Medical Sciences and Hospital Administration.

Vol. XXXVII.—No. 950. DECEMBER 10, 1904.

### THE TREATMENT OF CANCER.

The hope expressed by Mr. Mayo Robson, when delivering the Bradshaw Lecture on the 1st instant, that the substance of what he had said might filter through the profession to the public, and might serve to convince them that there is no salvation from cancer in quackery, will be generally shared by all who are acquainted with the facts of the case, and may well serve to illustrate the value of every opportunity which allows surgeons or physicians to address themselves, directly or indirectly, to a wider circle than that of the profession itself. We doubt whether the Bradshaw Lecture was intended to afford such an opportunity, and whether it was not designed for the instruction of purely scientific audiences in the latest developments of medicine or surgery; but we shall assuredly not quarrel with the departure from precedent, if such it were, of the latest occupant of the chair. It is eminently desirable that the public should be informed, by one of the most skilful and most successful of operating surgeons, not only that a large proportion of cases of cancer are curable, but also that the possibility of cure depends mainly upon the promptitude with which it is sought. It cannot be too widely known that "the watching of a doubtful tumour of the mammary gland until it becomes definitely malignant is an unjustifiable and blameworthy procedure," or that, in view of the frequency with which cancer is developed on the seat of some local irritation, many of the applications which have been made to growths of various kinds have probably been actively mischievous. It cannot be too widely known that any persistent local irritation, especially of a part of the body prone to cancer, is dangerous, or that, although "patients have usually themselves to blame in ignoring what seem to them rather trifling ailments, yet sometimes the medical attendant, to avoid frightening his client, makes light of a condition which ought not to be neglected." It must be conceded that it is better to "alarm and cure", than to create a false sense of security, leading to the postponement of an operation until its success can no longer be regarded as a certainty; and, generally speaking, practical surgeons will be at one with Mr. Mayo Robson in his declaration that although the methods of treatment which have been suggested, psychical, physical, medicinal, photo-therapeutic, electrical, etc., are almost too many to mention, yet that one and all of them, except in cases of rodent ulcer, have been found wanting, and, so far as our present knowledge is concerned, "the only hope of cure in malignant disease lies in early diagnosis and in immediate, complete, and wide removal." In these words we have, it cannot be questioned, the final verdict of surgery as guided by the knowledge and experience of to-day.

When Mr. Mayo Robson departed from the domain of surgery, and made an excursus into the domains of bacteriology and morbid histology, he was, we venture to think, somewhat less successful. His declaration that "our" present knowledge does not warrant such a positive statement as that of the able superintendent of the Cancer Research Fund, that from the histological character, method of growth, and absence of specific symptomatology it is not permissible to seek for the causative factor of cancer outside the life processes of the cells, is one with which Dr. Bashford himself may safely be left to deal. There is at least one important class of cancers in which no stage of prior local disturbance or irritation has ever been observed, and in which the invasion of the affected organ by any parasite is extremely improbable, the class known as gliomata of the retina. Such gliomata have been discovered within the first month of life, and are possibly always congenital, while their appearance is practically limited to the period of early childhood, and they are quite certainly original or primary growths. The ocular cancers of adult age, the melanotic or sarcomatous tumours of the choroid, are certainly often, and possibly always, secondary, and hence may have no bearing upon the argument, but it is certain that no definite "irritation" is an ordinary precursor of their appearance. With reference to irritation, Mr. Robson described two varieties of cancer as having become less frequent than formerly—cancer of the lip from the comparative disuse of clay-pipes, and chimney-sweep's cancer from the effects of legislation. We could not quite follow the latter statement, because the only legislation bearing upon chimney-sweeps with which we are acquainted was the prohibition of climbing-boys, while the special form of cancer did not so much affect children as adults. The adult chimney-sweep of to-day, as much as his predecessor, handles and carries large quantities of soot in fine powder, which must certainly make its way among all the interspaces of his clothing, and be just as liable to lodge in the wrinkles of his scrotum as ever it was. We apprehend that the special proclivity of his scrotum to cancer was due to the character of its surface, and to the facilities for the lodgment of soot which this character afforded, as well as to the comparative difficulty of cleansing it. There seems, in fact, to be some definite connection between soot irritation and cancer, for Mr. Robson mentioned a case in which the disease appeared on the forearm of a gardener, apparently as a consequence of frequently carrying a basket containing soot. If we consider that soot is carbon which has been volatilised at a high temperature, and deposited in a position apparently little favourable to the development of parasitic life, we shall probably conclude that the fact, if such it be, of its tendency to produce cancer is one that lends some degree of support to the conclusions set forth by Dr. Bashford, and disputed, as it seems to us on very slender and insufficient ground, by Mr. Mayo Robson. The ultimate decision of the question must be left to research and time.

